# Cardiac fibroblast activation and hyaluronan synthesis in response to hyperglycemia and diet-induced insulin resistance

**DOI:** 10.1038/s41598-018-36140-6

**Published:** 2019-02-12

**Authors:** Daniel J. Gorski, Anne Petz, Christina Reichert, Sören Twarock, Maria Grandoch, Jens W. Fischer

**Affiliations:** 1 Institut für Pharmakologie und Klinische Pharmakologie, University Hospital, Heinrich-Heine-University, Düsseldorf, Germany; 2 CARID, Cardiovascular Research Institute Düsseldorf, University Hospital, Heinrich-Heine-University Düsseldorf, Düsseldorf, Germany

## Abstract

Diabetic patients are at a greater risk of heart failure due to diabetic cardiomyopathy and worsened outcome post-myocardial infarction. While the molecular mechanisms remain unclear, fibrosis and chronic inflammation are common characteristics of both conditions. Diabetes mellitus (types I and II) results in excessive hyaluronan (HA) deposition *in vivo*, and hyperglycemia stimulates HA synthesis for several cell types *in vitro*. HA-rich extracellular matrix contributes to fibrotic, hyperplastic and inflammatory disease progression. We hypothesized that excessive hyperglycemia-driven HA accumulation may contribute to pathological fibroblast activation and fibrotic remodelling in diabetic patients. Therefore, we analysed the impact of both hyperglycemia and diet-induced obesity and insulin resistance on HA matrix formation and cardiac fibroblast activation. Here we report that cardiac fibroblasts isolated from mice on a diabetogenic diet acquire pro-fibrotic gene expression without a concomitant increase in HA matrix deposition. Additionally, hyperglycemia alone does not stimulate HA synthesis or cardiac fibroblast activation *in vitro*, suggesting that the direct effect of hyperglycemia on fibroblasts is not the primary driver of fibrotic remodelling in cardiac diabetic maladaptation.

## Introduction

Diabetes mellitus increases the risk of heart failure^1^ and worsens the outcome after myocardial infarction (MI)^2^. Although the mechanisms are not entirely clear, increased fibrosis^3^, the result of activated fibroblasts, appears to be aggravated in diabetic patients. Fibroblast activation refers to fibroblasts’ phenotypic transition from a resting state to an active state of heightened proliferation, migration, contractility and extracellular matrix production^4^. The most widely used markers for identifying activated fibroblasts are α-smooth muscle actin (α-SMA) and collagen expression. Originally thought to be responsible only for scar formation, cardiac fibroblast function is now known to be multifaceted. In addition to being the primary matrix-producing cells in the myocardium, fibroblasts secrete pro-inflammatory factors in response to injury^5^ and mediate cardiomyocyte survival^6^. Understanding the mechanisms behind fibroblast activation will help us identify factors that promote maladaptive cardiac healing.

Though cardiac fibroblast metabolism is not fully understood, it is generally well established that hyperglycemic conditions induce an activated fibroblast phenotype, in which several signalling mechanisms have been proposed involving: STAT2/3^7^, ERK1/2^8^, Rho/ROCK^9^, intracellular RAAS^10^, PAR-4^11^ and NLRP3 inflammasome activation^12^.

We have previously reported that the extracellular glycosaminoglycan hyaluronan (HA) plays a role in activating cardiac fibroblasts after MI^13^. HA is also known to facilitate lung^14,15^ and dermal^16^ fibroblast activation. HA synthesis is carried out by HA synthases 1, 2 and 3 (HAS1–3), which are integrated into the plasma membrane and elongate a repeating disaccharide chain of (glucuronic acid-β1, 3-N-acetylglucosamine-β1, 4-). To achieve this, HASs draw from a cytosolic pool of nucleotide sugar precursors synthesised by the hexosamine biosynthesis pathway, an alternative glucose metabolism pathway. Thus, HA formation is connected to glucose metabolism and is therefore susceptible to dysregulation in disease states such as diabetes.

In fact, HA accumulation during the progression of types I and II diabetes mellitus has been documented in human aortas^17^, pancreatic islets, lymphoid tissues^18^ and circulation^19^. Animal models of diabetes also show excessive HA deposits, namely in the kidneys of streptozotocin-treated rats^20^, the skeletal muscles of mice on a high-fat diet^21^ and the pancreatic islets of the DORmO mouse^22^. Further, multiple cell types are known to stimulate HA synthesis in response to hyperglycemia. These include, renal proximal tubular epithelial cells^23^, renal and skin fibroblasts^24,25^, vascular smooth muscle cells^26^, endothelial cells^27^, mesangial cells^28^ and oesophageal cancer cells^29^. The mechanism behind this stimulation involves the higher availability of sugar precursors for HA synthesis as well as heightened β-linked N-acetylglucosamine (O-GlcNAc) modifications that mediate the transcriptional and post-translational regulation of HAS2, which in turn facilitates HA production^30,31^.

Because it accumulates in a number of organs during diabetes and is known to facilitate fibroblast activation, HA may be a contributing factor in the fibrotic remodelling typical of diabetic cardiac pathology and cardiac maladaptation post-MI. Therefore, we investigated the possible link between HA matrix accumulation and cardiac fibroblast activation in response to hyperglycemia *in vitro* and diet-induced insulin resistance *in vivo*.

## Results

### Hyperglycemia alone does not stimulate HA synthesis or activation in cardiac fibroblasts

**Figure 1:**
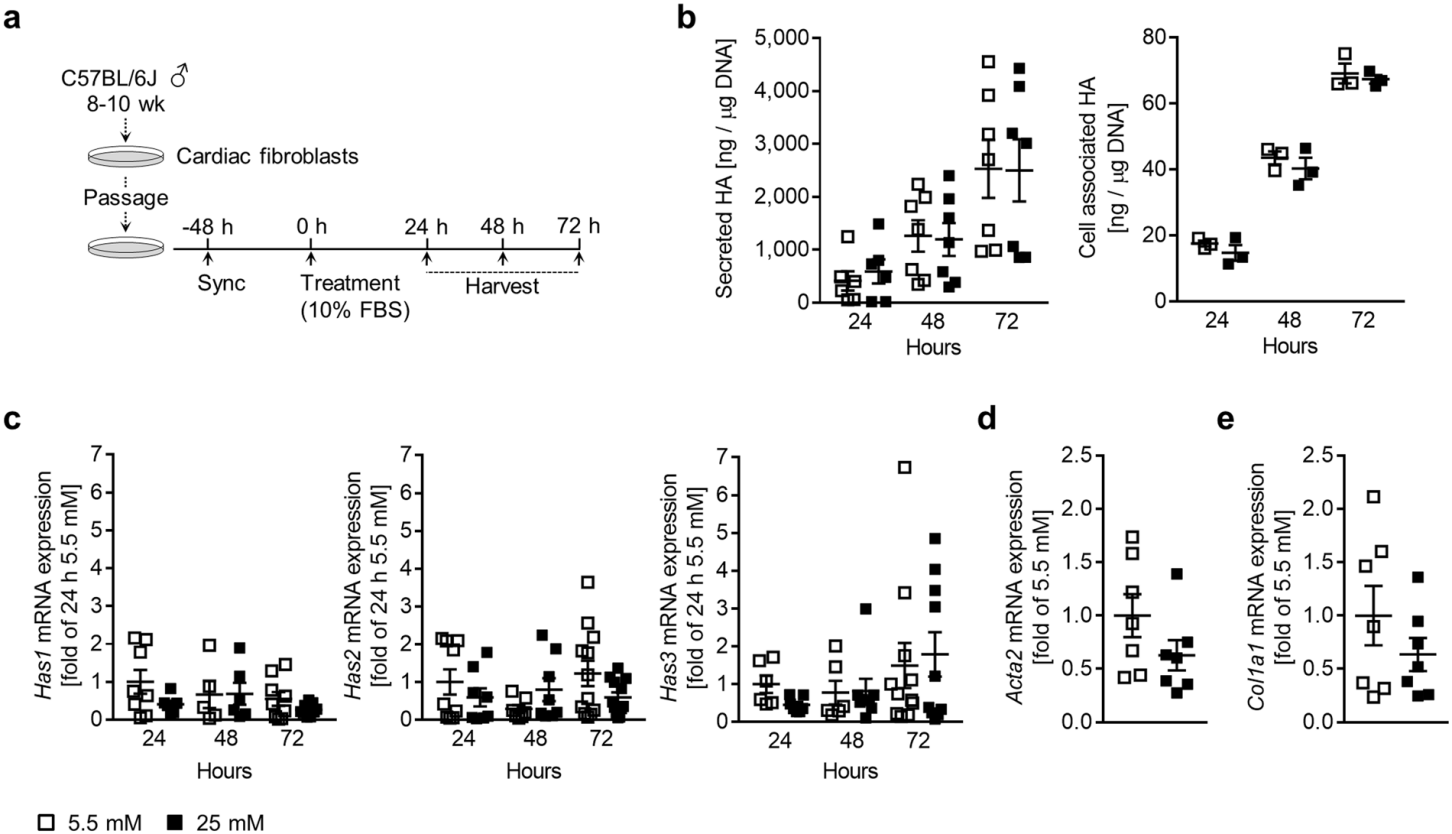
Hyperglycemia does not augment either HA matrix production or cardiac fibroblast activation *in vitro*. Cultures of primary cardiac fibroblasts were treated with media containing 5.5 or 25 mM glucose in 10% FBS for up to 72 hours. (**a**) Experimental design schematic. (**b**) Quantification of HA secretion into the media (n = 7) and deposition into the cell layer (n = 3). (**c**) Quantification of *Has1, 2* and *3* mRNA expression, expressed as fold relative to 24 h 5.5 mM, 24 h (n = 6–9), 48 h (n = 5–8) and 72 h (n = 9–12). (**d**) Quantification of *Acta2* mRNA expression after 72 h, expressed as fold of 5.5 mM (n = 7). (**e**) Quantification of *Col1a1* mRNA expression after 72 h, expressed as fold of 5.5 mM (n = 7). For analysis of mRNA expression, *Rn18S* was used as an internal control. Data represent mean ± SEM; one-way ANOVA with Sidak’s multiple-comparison correction (**b**,**c**) and unpaired t-test (**d**,**e**).

Primary cardiac fibroblasts cultures isolated from 8–10-week-old male C57BL/6 J mice were proven by flow cytometry to be 96% pure (Supplemental Fig. 1). To test the effects of hyperglycemia on HA production and fibroblast activation, cells were incubated in media containing 10% FBS and 5.5 mM (normoglycemia) or 25 mM (hyperglycemia) glucose for up to 72 h (Fig. 1a). HA deposition into the media and cell layer was quantified after 24, 48 and 72 h using an HA-binding protein-based test kit (Fig. 1b). The vast majority of the total measured HA was secreted into the media (95–97%) and a significant increase of HA deposition was detected daily, suggesting a continual deposition with little or no turnover. Surprisingly, there was no significant difference in the amount of HA synthesised between normoglycemic and hyperglycemic cultures at any of the time points analysed. The impacts of osmotic pressure and glutamine concentration also showed no differences (Supplemental Fig. 2). Further, we observed no significant regulation of *Has1, 2* or *3* mRNA expression (Fig. 1c). The expression of *Acta2* (α-smooth muscle actin, α-SMA) and *Col1a1* (collagen type I) mRNAs, markers of cardiac fibroblast activation, were also unchanged (Fig. 1d,e). In fact, both markers displayed a trend towards lower expression in hyperglycemic conditions.

It has been previously shown in mesangial cells that excessive HA matrix formation is cell cycle dependent, occuring only when dividing cells are challenged with hyperglycemia^20^. Therefore, HA synthesis was quantified in actively proliferating cardiac fibroblasts; again, hyperglycemic conditions had no effect on proliferation or the amount of HA synthesised (Supplemental Fig. 2). Separate reports also indicate that chronic exposure to hyperglycemia might be necessary to induce changes in HA abundance^26^. Accordingly, cardiac fibroblasts were cultured continuously in normoglycemic or hyperglycemic media for three passages, and no significant difference in HA synthesis was observed (Supplemental Fig. 2).

### Hyperglycemia reduces TGF-β1 stimulation

**Figure 2:**
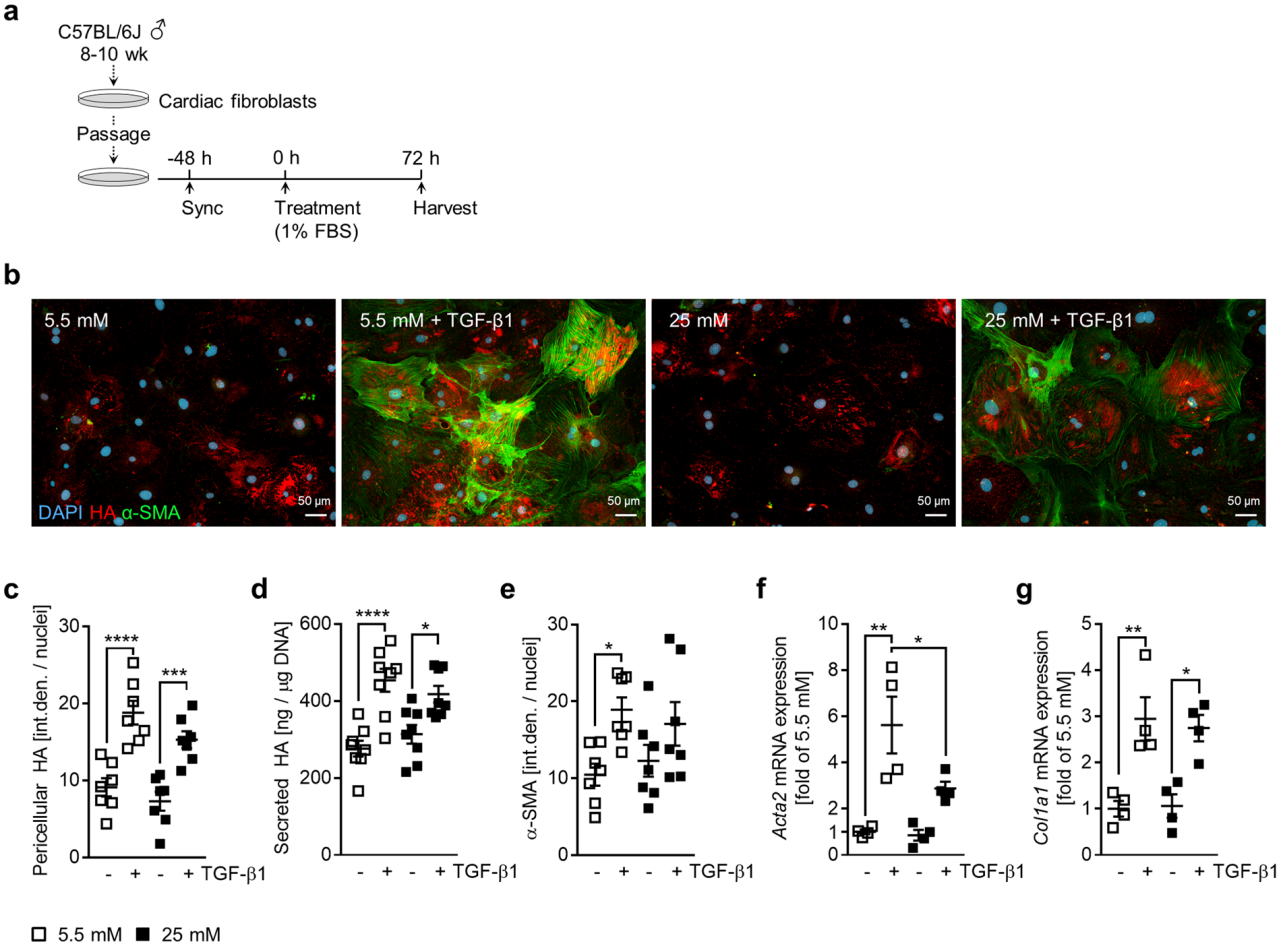
Hyperglycemia does not enhance the HA matrix production or activation of cardiac fibroblasts stimulated with TGF-β1. Cultures of primary cardiac fibroblasts were treated with media containing 5.5 or 25 mM glucose in 1% FBS ± 10 ng/mL TGF-β1 for 72 hours. (**a**) Experimental design schematic. (**b**) Representative images of immunocytochemical staining of HA (red) and α-SMA (green) with quantification (**c**,**e**) (n = 7). (**d**) Quantification of HA secretion into the media (n = 8). (**f**) Quantification of *Acta2* mRNA expression, expressed as fold of 5.5 mM without TGF-β1 (n = 4). (**g**) Quantification of *Col1a1* mRNA expression, expressed as fold of 5.5 mM without TGF-β1 (n = 4). For analysis of mRNA expression, *Rn18S* was used as an internal control. Data represent mean ± SEM; one-way ANOVA with Sidak’s multiple-comparison correction (**c**,**d**,**e**,**f**,**g**). *P ≤ 0.05, **P ≤ 0.005, ***P ≤ 0.0005, ****P < 0.0001.

To determine if hyperglycemia affects growth factor-driven HA synthesis and fibroblast activation, cultures were stimulated with TGF-β1, a potent inducer of both phenomena, in the presence of 1% FBS and either 5.5 or 25 mM glucose for 72 h (Fig. 2a). Immunocytochemical stainings were performed to visualise and quantify the HA pericellular matrix as well as α-SMA expression (Fig. 2b). Quantification of pericellular HA (Fig. 2c), as well as secreted HA (Fig. 2d), showed significant stimulation upon TGF-β1 treatment in normoglycemic and hyperglycemic conditions. TGF-β1 stimulation also induced α-SMA expression and stress fibre formation in normoglycemic cultures, though to a lesser extent under hyperglycemic conditions (Fig. 2e). Similarly, *Acta2* mRNA expression in hyperglycemic cultures demonstrated a weaker response to TGF-β1 (Fig. 2f). TGF-β1 stimulation resulted in significantly elevated *Col1a1* mRNA expression in both glucose concentrations (Fig. 2g). There were no significant differences at baseline (without TGF-β1) in any of the assays performed.

### Glucose transport is not elevated by hyperglycemia

**Figure 3:**
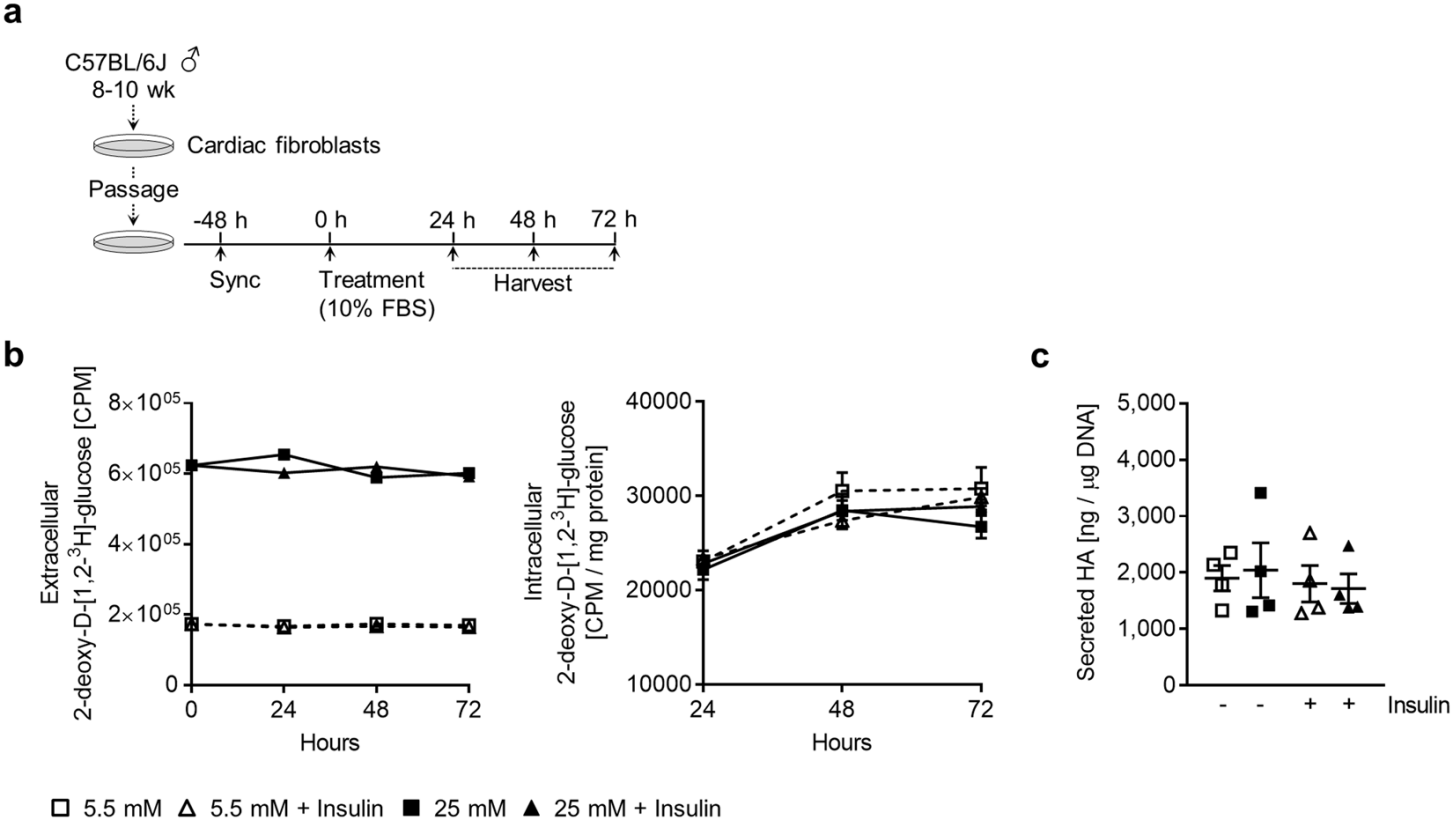
Hyperglycemia does not augment the glucose uptake of cardiac fibroblasts *in vitro*. Cultures of primary cardiac fibroblasts were treated with media containing 5.5 or 25 mM glucose supplemented with radiolabeled 2-deoxyglucose in 10% FBS for up to 72 hours ± 100 nM insulin. (**a**) Experimental design schematic. (**b**) Time course of radiolabeled glucose uptake (n = 4). (**c**) Quantification of HA secretion into the media (n = 4). Data represent mean ± SEM; two-way ANOVA with Sidak’s multiple-comparison correction (**b**) and one-way ANOVA with Sidak’s multiple-comparison correction (**c**).

To test whether hyperglycemic media results in an elevated transport of glucose across the cell membrane of cardiac fibroblasts and whether this is an insulin-sensitive process (as is the case with cardiomyocytes^32^), we quantified the uptake of a radiolabeled glucose analogue. Cultures were given either 5.5 or 25 mM glucose media supplemented with an equal proportion of radiolabeled 2-deoxyglucose, 0.2 µCi/ml and 0.9 µCi/ml 2-deoxy-D-[1, 2-^3^H]-glucose, respectively; some cultures were given additional insulin (Fig. 3a). Uptake was quantified at 24, 48 and 72 h. Surprisingly, we observed no difference in the amount of intracellular radioactive 2-deoxyglucose between 5.5 mM or 25 mM treated groups at any time point (Fig. 3b). Furthermore, over 72 h, cardiac fibroblasts transported very little glucose: 30785 CPM/mg protein ± 2252, n = 4 (5.5 mM condition) and 26741 CPM/mg protein ±1197, n = 4 (25 mM condition), which corresponded to only 2.7% and 0.75% of the total extracellular radiolabeled analogue, respectively. Insulin supplementation had no effect on glucose transport at any of the time points analysed. However, acute insulin stimulation did result in Akt phosphorylation and notably elevated the extracellular acidification rate (ECAR) (Supplemental Fig. 3), suggesting that cardiac fibroblasts do respond to insulin. Though, this was not accompanied by any significant increase in glucose uptake. This was further supported by the much greater abundance of *Slc2a1* (GLUT1) (98.9% ± 0.62, n = 4) mRNAs than *Slc2a4* (GLUT4) (0.997% ± 0.61, n = 4) mRNAs (Supplemental Fig. 3). 5.5 and 25 mM glucose-treated groups supplemented with insulin also demonstrated no alteration in HA production (Fig. 3c).

### Acute hyperglycemia alters glucose utilisation

To examine whether hyperglycemic medium affects cellular metabolism despite the absence of substantially elevated glucose transport, we assayed typical glucose utilisation pathways (Supplemental Fig. 4). Cultures were acutely exposed to normoglycemic or hyperglycemic media for 1 h, after which the extracellular rate of acidification (ECAR) and oxygen consumption rate (OCR) were quantified in real time to estimate glycolysis and mitochondrial oxidation, respectively. Hyperglycemia resulted in significantly elevated ECAR and moderately reduced OCR, suggesting the acute exposure to hyperglycemia stimulated glycolysis while suppressing mitochondrial respiration. However, extracellular lactate levels were not significantly altered after 72 h, indicating that the increased glycolysis eventually normalises. Additionally, hyperglycemia raised both glycogen content and O-GlcNAcylated protein levels, as measured after 72 h. Taken together, these data indicate that hyperglycemic conditions may alter certain aspects of glucose utilisation without a marked difference in total transport. Inhibiting glycolysis has been previously shown to stimulate HA synthesis, presumably making more sugar precursors available to the hexosamine biosynthesis pathway^29^. To examine whether blocking either glycolysis or glycogen storage could reroute sugar precursors to HA synthesis in cardiac fibroblasts, cultures were treated with siRNAs to knock down phosphofructokinase (*Pfkm*) or glycogen synthase (G*ys1*) expression. We observed no significant differences in HA synthesis between normoglycemic and hyperglycemic cultures in any of the conditions tested.

### Cardiac fibroblasts have high metabolic flexibility

**Figure 4:**
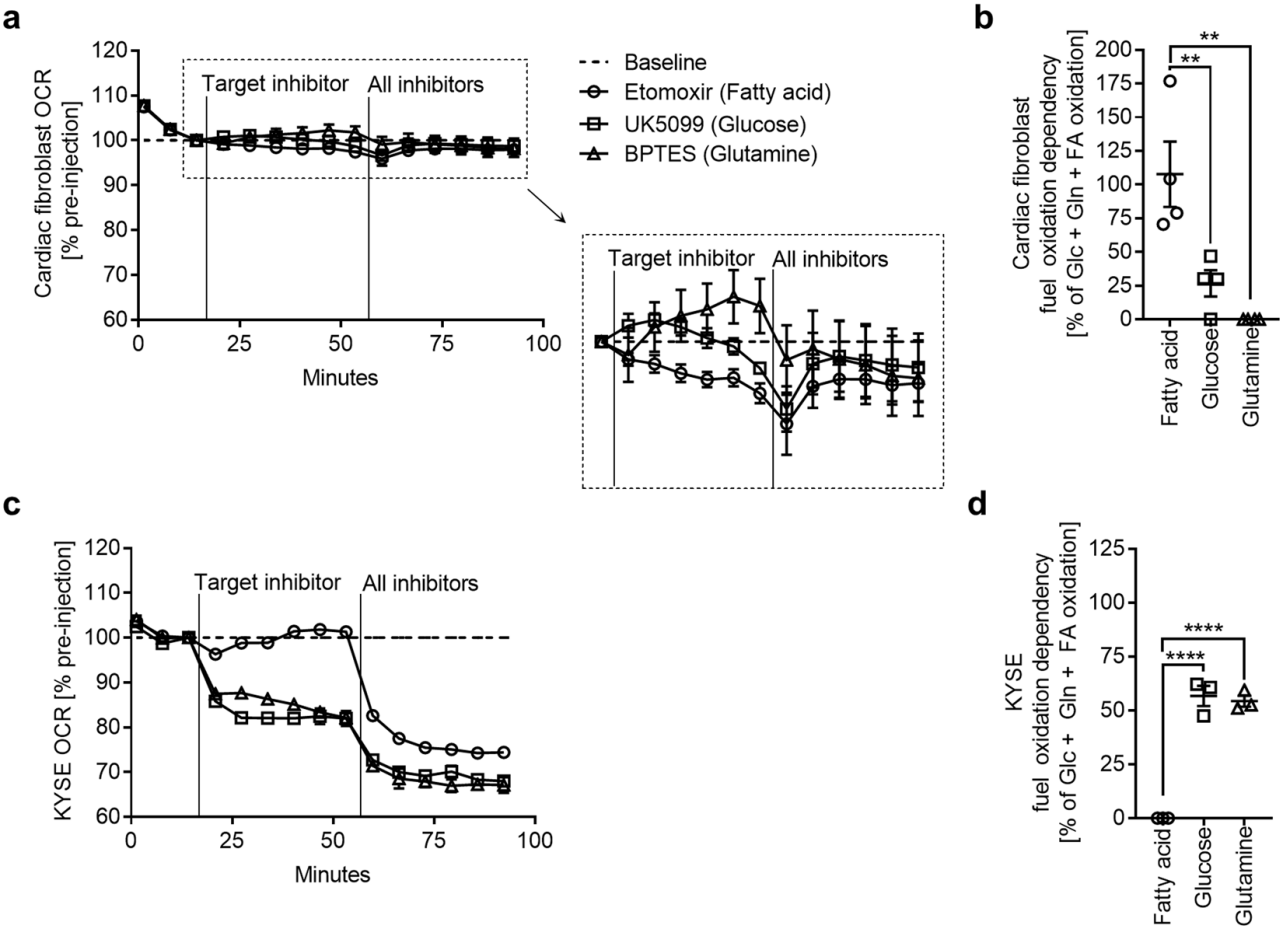
Cardiac fibroblasts exhibit high metabolic flexibility. To block the mitochondrial oxidation of glucose, glutamine and fatty acids, cultures of primary cardiac fibroblasts and oesophageal cancer cells (KYSE) were given the inhibitors UK5099, BPTES and etomoxir, respectively. Using a Seahorse XFe96 Analyzer, the oxygen consumption rate (OCR) was monitored in real time while injections of single pathway inhibitors were administered (Target inhibitor), followed by an injection of the remaining 2 inhibitors (All inhibitors) to determine each pathway’s contribution to the total mitochondrial respiration. (**a**) OCR time course of cardiac fibroblasts, values displayed relative to pre-injection OCR baseline (n = 4). (**b**) Relative cardiac fibroblast substrate dependency (n = 4). (**c**) OCR time course of KYSE cells, values displayed relative to pre-injection OCR baseline (n = 3). (**d**) Relative KYSE cell substrate dependency (n = 3). Data represent mean ± SEM; one-way ANOVA with Sidak’s multiple-comparison correction (**b**,**d**). **P ≤ 0.005, ****P < 0.0001.

To decipher the surprising lack of elevated glucose uptake and HA synthesis in cardiac fibroblasts exposed to hyperglycemia, we performed a mitochondrial fuel dependency assay. We used oesophageal cancer cells (KYSE) for comparison, as they are known to have elevated intracellular UDP-glucose and subsequently stimulate HA synthesis in response to hyperglycemia^29^. Mitochondrial respiration was measured in real time, while specific inhibitors of the major oxidative pathways were administered alone and in combination. These inhibitors were UK5099 to inhibit pyruvate shuttling into the mitochondria (glucose oxidation), Bis-2-(5-phenylacetamido-1,3,4-thiadiazol-2-yl) ethyl sulphide (BPTES) to inhibit glutamate formation (glutamine oxidation) and etomoxir to inhibit long-chain fatty acid shuttling into the mitochondria (fatty acid oxidation). Strikingly, the mitochondrial respiration of cardiac fibroblasts was largely unaffected by any of the inhibitors alone or in combination, and full inhibition was only able to reduce cellular respiration by 2–3% (Fig. 4a); presumably, fibroblasts oxidised ketone bodies during this time^33^. Out of the three pathways, etomoxir administration to inhibit long chain fatty acid shuttling induced the most significant reduction of OCR, indicating a relatively greater dependence on fatty acid oxidation than glucose or glutamine (Fig. 4b). KYSE cells showed 30% less respiration with full inhibition under the same conditions (Fig. 4c) and were highly dependent on glucose and glutamine oxidation (Fig. 4d). These results suggest that cardiac fibroblasts have a highly flexible metabolism and can function independently of glucose oxidation.

### Diabetogenic diet promotes cardiac fibroblast activation without excessive HA accumulation

**Figure 5:**
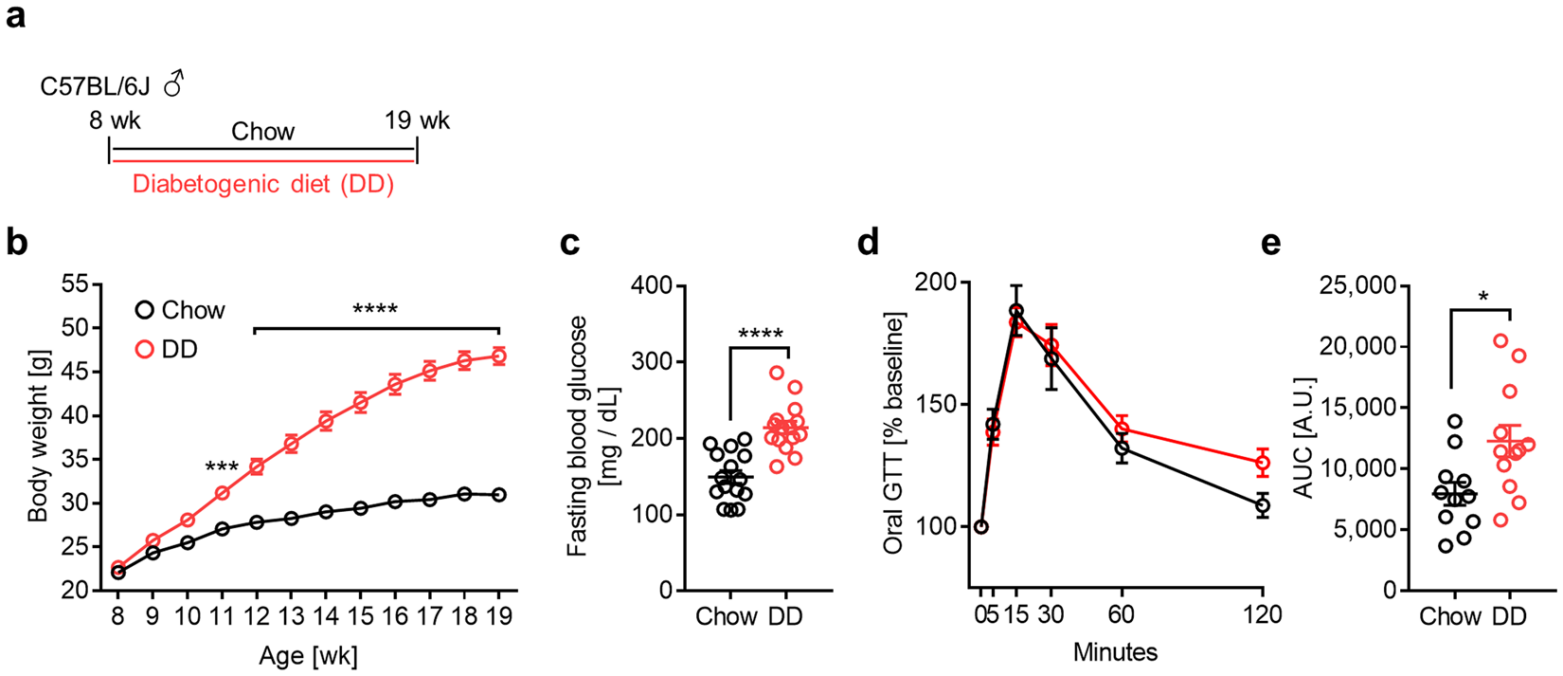
Model of diet-induced obesity and insulin resistance. 8-week-old male C57BL/6 J mice were fed a standard chow (chow) or diabetogenic diet (DD) for 11 weeks. (**a**) Feeding schematic. (**b**) Body weight (n = 15). (**c**) Fasting blood glucose (n = 15). (**d**) Fixed-dose oral glucose tolerance (n = 11,12) with area under the curve (AUC) quantification. (**e**) Data represent mean ± SEM; two-way ANOVA with Sidak’s multiple-comparison correction (**b**) and unpaired t-test (**c**,**e**). *P ≤ 0.05, ***P ≤ 0.0005, ****P < 0.0001.

**Figure 6:**
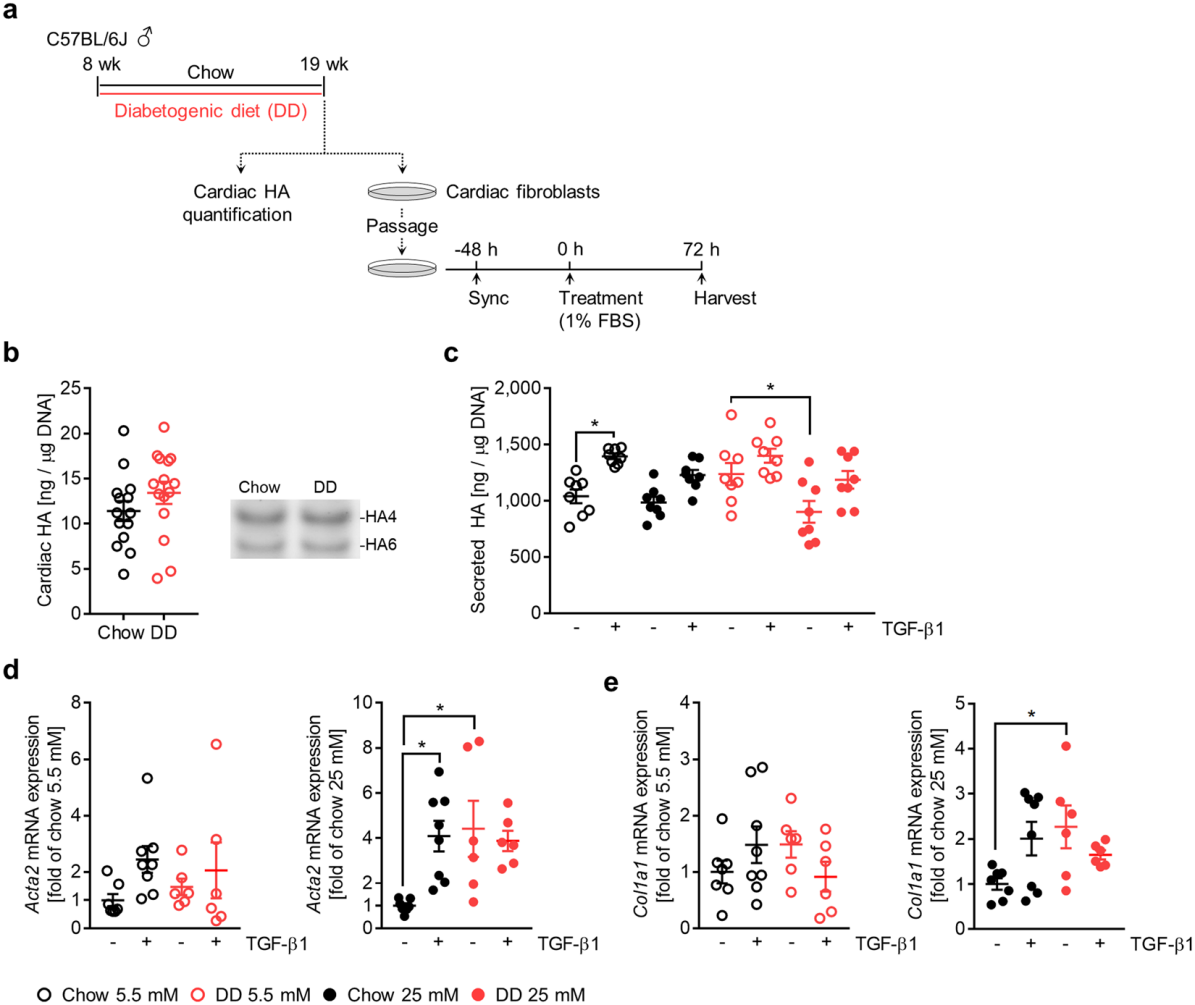
Diabetogenic diet does not result in excessive HA accumulation but does promote cardiac fibroblast activation. 8-week-old male C57BL/6J mice were fed standard chow (chow) or diabetogenic diet (DD) for 11 weeks. Subsequently, cardiac HA content was quantified, and cardiac fibroblasts were isolated and treated with media containing 5.5 or 25 mM glucose in 1% FBS ± 10 ng/mL TGF-β1 for 72 hours. (**a**) Feeding and experimental schematic. (**b**) Quantification and representative gel of cardiac HA assessed by FACE (n = 15). (**c**) Quantification of HA secretion by isolated cardiac fibroblasts (n = 8). (**d**) Quantification of *Acta2* mRNA expression of isolated cardiac fibroblasts, expressed as fold of chow-fed without TGF-β1 (n = 6–8). (**e**) Quantification of *Col1a1* mRNA expression of isolated cardiac fibroblasts, expressed as fold of chow-fed without TGF-β1 (n = 6–8). For analysis of mRNA expression, *Rn18S* was used as an internal control. Data represent mean ± SEM; unpaired t-test (**b**) and one-way ANOVA with Sidak’s multiple-comparison correction (**c**,**d**,**e**). *P ≤ 0.05.

To further investigate cardiac fibroblast HA matrix formation and activation, we employed a model of diet-induced obesity and insulin resistance in which 8-week-old male C57BL/6 J mice were given standard chow (chow) or a diabetogenic diet (DD) for 11 weeks^34^ (Fig. 5a). Mice on a diabetogenic diet exhibited increased body weight gain (Fig. 5b), elevated fasting blood glucose (Fig. 5c) and impaired glucose tolerance (Fig. 5d,e). At 19 weeks of age, the total cardiac HA content was quantified and cardiac fibroblasts were cultured to determine their HA production and activation status in response to TGF-β1 and hyperglycemia (Fig. 6a). Analysing the cardiac HA matrix *in vivo* by fluorophore-assisted carbohydrate electrophoresis (FACE) revealed no differences between chow- and DD-fed mice (Fig. 6b). Similarly, cardiac fibroblasts isolated from chow-fed mice produced the same amount of HA as DD-fed mice, whether the fibroblasts were cultured in normoglycemic or hyperglycemic media (Fig. 6c). By comparison, cardiac fibroblasts isolated from chow-fed mice and cultured in normoglycemic conditions displayed the strongest stimulation of HA synthesis in response to TGF-β1. Interestingly, fibroblasts isolated from DD-fed mice and cultured in hyperglycemic media had significantly lower HA synthesis at baseline than fibroblasts isolated from DD-fed mice cultured in normoglycemia. Analysing fibroblast activation revealed that cardiac fibroblasts isolated from DD-fed mice had higher *Acta2* (Fig. 6d) and *Col1a1* (Fig. 6e) baseline expressions and had a diminished response to TGF-β1 when stimulated in hyperglycemic media. This was also observable in normoglycemic conditions but to a lesser extent.

## Discussion

By investigating a possible link between HA synthesis and cardiac fibroblast activation in response to hyperglycemia and in a model of obesity and insulin resistance, we demonstrated that excessive HA matrix formation is not required for cardiac fibroblast activation. These data also demonstrate that hyperglycemia alone is not enough to activate adult murine cardiac fibroblasts. However, using a model of obesity and insulin resistance, in which mice are given a diabetogenic diet for 11 weeks, we observed that cardiac fibroblasts acquire enhanced myofibroblastic/fibrotic gene expression and reduced responsiveness to TGF-β1. Similar results have been recently reported, in which cardiac fibroblasts isolated from db/db mice exhibit elevated collagen synthesis and a weakened TGF-β1 response^35,36^. Our results support the argument that fibrosis in diabetic cardiomyopathy is not solely mediated by hyperglycemia’s direct effects on cardiac fibroblasts. Many other aspects of diabetic pathology are able to activate cardiac fibroblasts^37^ including: TNF-α, angiotensin II, TGF-β1, oxidative stress, elevated monocyte infiltration^38^ and increased macrophage abundance^39^. Another interpretation might suggest that acute hyperglycemia (72 h) is not enough to generate fibrotic effects, whereas chronic exposure, via diabetogenic diet or otherwise, sensitises resident cardiac fibroblasts to activation.

Numerous investigations (>50 publications to date) into hyperglycemia’s influence on cardiac fibroblast phenotype have generally concluded that hyperglycemia induces cardiac fibroblast activation, i.e. enhanced proliferation, collagen synthesis and α-SMA expression^7,10,40,41^. Our results do not fully align with these findings, as we have demonstrated that ‘healthy’ cardiac fibroblasts do not transport additional glucose when given an extracellular overabundance and do not experience enhanced proliferation or increased collagen or α-SMA expression. It is worth noting that most of the published literature on this subject uses neonatal rat and neonatal mouse cardiac fibroblasts, whereas we used 8+-week-old mice. In fact, separate work using adult murine cardiac fibroblasts also found that glucose concentration did not affect activation status^36^. Interestingly, neonatal cardiac fibroblasts, unlike the cells we examined, are insulin sensitive and express GLUT4^42^. Moreover, foetal/neonatal hearts primarily utilise glucose and lactate oxidation, while adults primarily utilise fatty acids^43–45^. Therefore, the metabolic substrate preference between neonatal and adult cardiac fibroblasts is likely very different, and may explain their distinct reactions to hyperglycemia.

Our results also demonstrate that adult cardiac fibroblasts have more abundant *Slc2a1* (GLUT1) mRNAs to *Slc2a4* (GLUT4) mRNAs, indicating their glucose uptake is mostly insulin-independent; this was confirmed by glucose uptake assay. Since the most abundant glucose transporter in the adult heart is GLUT4^46^, cardiac fibroblasts have inherently different glucose regulation than their neighbouring cells.

Additionally, our data show that murine cardiac fibroblasts can withstand hyperglycemia without generating an excessive HA matrix. This result contrasts many previous studies in other cell types that demonstrate hyperglycemic media increases HA production and results in more abundant *Has* mRNA transcripts^23–28,47–50^. However, because hyperglycemia did not alter glucose transport across the cell membrane of the cardiac fibroblasts used here, no additional sugar substrates would be available for hexosamine biosynthesis. *Has* mRNA expression was also unchanged; therefore, it is not surprising that HA production remains equal to that in normoglycemic controls.

We observed signs of altered glucose utilisation in which hyperglycemia may drive glycolysis, glycogen formation and increased cellular O-GlcNAc modifications. Although they contradict the equal uptake of radiolabeled glucose, these findings may be attributed to either acute effects, which eventually normalise over 72 h, or may represent minimal changes in glucose uptake that were too small to detect by glucose uptake assay. It should be noted that the overall extracellular lactate levels were unchanged at 72 h, and the increase in O-GlcNAc protein modifications was not as pronounced as others reports^51^. It is likely that a minimal increase in glucose transport occurs acutely but is too small to noticeably affect HA synthesis, as the hexosamine biosynthesis pathway is thought to utilise only 2–5% of the available intracellular glucose^52,53^.

Much of the research on hyperglycemic stimulation of HA synthesis has used mesangial cells^20,28,54^. These studies showed that dividing cells pathologically activate HAS enzymes intracellularly in response to hyperglycemic stress, and the resulting HA matrix is extruded after cell division. In our experiments, dividing cardiac fibroblasts did not stimulate HA production in either intra- or extracellular compartments under hyperglycemic stress. However, the reports mentioned above indicate mesangial cells have a doubling time of roughly 24 h^20^, whereas the cardiac fibroblasts we examined took nearly 65 h to complete one doubling. This phenotypic trait could be responsible for the different responses to hyperglycemia, as cardiac fibroblasts may be too quiescent to experience the same phenomenon *in vitro*.

We believe the ability of cardiac fibroblasts to resist hyperglycemia is an interesting characteristic that needs further investigation. The experiments presented in Fig. 4 demonstrate that cardiac fibroblasts are highly flexible in utilising metabolic substrates and have a relatively greater dependence on fatty acid oxidation than glucose oxidation, similar to the adult heart as a whole^43^. Furthermore, cardiac fibroblasts used only a small percentage of their glucose over 72 h, which is in keeping with a previous report of human cardiac myofibroblasts using only 1% of available glucose over 96 h^55^. High metabolic flexibility and a preference for fatty acids may explain why the cells did not use their extra glucose supply, namely in the presence of FBS, which provides ample fatty acid substrates. Interestingly, KYSE cells, which accumulate HA under hyperglycemia^29^, were shown to be relatively inflexible and more dependent on glucose oxidation for mitochondrial respiration. These cell types’ two distinct metabolisms could underlie their dissimilar stimulation of HA synthesis in response to hyperglycemia. In other words, dependence on glucose oxidation and/or metabolic inflexibility may be a common characteristic of cells that create excessive HA matrices under pathological conditions.

While HA matrix formation did not correlate with cardiac fibroblast activation in our study, nor did cardiac HA accumulate under diabetic conditions, excessive HA matrix formation and its contribution to fibrotic remodelling in diabetic patients cannot be completely ruled out, especially in the post-ischemic setting. We have previously demonstrated robust HA formation in the acute phase post-MI^13^, while the uninjured heart has a relatively minimal HA matrix. Further, failing myocardium is known to take on a foetal gene program^56,57^ and go through metabolic reprogramming^58^, which includes elevated glucose uptake^59^. Each of these changes has the potential to modulate HA matrix production in response to hyperglycemia. Therefore, further investigation is needed into how cardiac disease states may affect HA synthesis in diabetic conditions.

## Methods

### Experimental animals

All mice used in this study were C57BL/6 J obtained from Janvier Labs. All experimental protocols were approved and performed in accordance with relevant guidelines and regulations set forth by the Landesamt für Natur, Umwelt und Verbraucherschutz (LANUV) Nordrhein-Westfalen, Bezirksregierung Düsseldorf, Aktenzeichen: 84-02.04.2015.A322.

### Diabetogenic diet

8-week-old male mice were fed a standard chow (chow) or diabetogenic diet (DD) (S7200-E010, EF Bio-Serv F1850; 24% sucrose, 35.85% lard, Ssniff) for 11 weeks to induce obesity and insulin resistance. Body weight was measured weekly. Fasting blood glucose and oral glucose tolerance were assayed at 19 weeks of age, after a 6 h fasting period. Glucose tolerance was tested with a fixed dose (orally administrered) of 2 mg glucose/mean kg body weight of chow-fed animals. Blood glucose was measured with the ACCU-CHEK Compact Plus device (Roche).

### Cell culture

Primary cardiac fibroblasts were isolated from 8–10-week-old or 19-week-old male mice via retrograde perfusion with collagenase type I (Worthington). The resulting cell suspension was filtered through 100 µm and 40 µm sterile filters, pelleted at 300 × *g* for 5 min, resuspended and plated in 100 mm dishes. After 4 h, non-adherent cells were removed by aspiration and the adherent cells were given fresh growth media consisting of 5.5 mM glucose DMEM supplemented with 10% FBS, 100 U/ml penicillin and 100 µg/ml streptomycin (all reagents from Gibco Life Technologies). After 48 h, the cell layers were washed with media to remove loosely adherent debris and then given 48 h further incubation in fresh growth media. Fibroblasts were then trypsin passaged and plated for experiments. Before treatment, cells were synchronised for 48 h in 1% FBS. Treatments included media containing 5.5 mM and 25 mM glucose, 100 nM insulin (Sigma-Aldrich) and 10 ng/mL TGF-β1 (Peprotech). Oesophageal cancer cells (KYSE) were grown in RPMI supplemented with 10% FBS, 100 U/ml penicillin and 100 µg/ml streptomycin as described previously^29^.

### Immunocytochemistry

Cardiac fibroblasts were seeded on glass coverslips and fixed using 70% ethanol, 5% acetic acid and 3.7% formaldehyde in PBS. HA affinity histochemistry was done with biotinylated HA-binding protein (Calbiochem) and Streptavidin-Cy3 (Invitrogen). α-SMA was detected with anti-α-smooth muscle actin (ab5694, Abcam) followed by an Alexa Fluor 647 goat anti-rabbit IgG (H + L) secondary antibody (Life Technologies); nuclei were stained with Roti-Mount Fluor-Care DAPI (Carl Roth). 180 images were captured for each culture using a Zeiss AxioObserver.Z1 microscope and stitched together using ZEN 2 software (Carl Zeiss Microscopy). The fluorescent signal was quantified from the resulting mosaic and normalised to the number of nuclei using ImageJ software (NIH).

### Hyaluronan quantification

To quantify HA in cell culture supernatants and cell layers, an HA-binding protein-based test kit (Corgenix Medical) was used. Before analysis, supernatants were spun at 300 × g for 5 min to remove floating debris. Cell layers were washed once with PBS and digested for 2 h at 60 °C with 1 mg/ml proteinase K (Invitrogen) in 100 mM ammonium acetate and 0.01% sodium dodecyl sulphate, followed by incubation at 100 °C to inactivate proteases. Values were normalised to DNA content determined by the Quant-iT PicoGreen dsDNA Assay Kit (Molecular Probes). To quantify HA in cardiac tissue, fluorophore-assisted carbohydrate electrophoresis (FACE) was used, as described previously^60^. Briefly, hearts were perfused with PBS heparin, harvested into cold PBS and washed twice on ice. The tissue was minced into small pieces, placed on a 100 um filter and washed with 10 mL PBS to remove residual blood. The tissue was then digested in proteinase K at 60 °C for 3 h, and glycosaminoglycans were isolated by ethanol precipitation and digested with Streptomyces hyaluronidase to generate HA tetramers (HA4) and hexamers (HA6). The resulting HA oligosaccharides were incubated with 2-aminoacridone (AMAC, Thermo Fischer Scientific) and separated by gel electrophoresis alongside an HA standard (LifeCore Biomedical) digested in the same fashion. Gel images were captured using a UVP ChemStudio imaging system (Analytik Jena) and quantified using Visionworks software (Analytik Jena). Values were normalized to DNA content as above.

### Quantitative real-time RT-PCR

RNA was extracted with PeqGOLD TriFast (PEQLAB Biotechnologie). cDNA was synthesised from 1 µg RNA using the QuantiTect Reverse Transcription Kit (Qiagen). Quantitative real-time RT-PCR was performed with Platinum SYBR Green qPCR SuperMix-UDG (Life Technologies) on a StepOnePlus Real Time PCR System (Applied Biosystems). Relative mRNA expression was calculated using the 2^−ΔΔC(t)^ method with *Rn18s* as an internal control. For direct comparison, mRNA expression of *Slc2a1* and *Slc2a4* was primer efficiency corrected as described previously^61^. The primer sequences used are as follows:

*Acta2*, forward 5′-CAGGCATGGATGGCATCAATCAC-3′, reverse 5′-ACTCTAGCTGTGAAGTCAGTGTCG-3′; *Col1a1*, forward 5′-CCCTGGTCCCTCTGGAAATG-3′, reverse 5′-GGACCTTTGCCCCCTTCTTT-3′; *Has1*, forward 5′-TATGCTACCAAGTATACCTCG-3′, reverse 5′-TCTCGGAAGTAAGATTTGGAC-3; *Has2*, forward 5′-CAAAAATGGGGTGGAAAGAG-3′, reverse 5′-ACAGATGAGGCAGGGTCAAG-3′; *Has*3, forward 5′-CTCAGTGGACTACATCCAGG-3′, reverse 5′- GACATCTCCTCCAACACCTC-3′; *Slc2a1*, forward 5′-GCATCTTCGAGAAGGCAGGT-3′, reverse 5′-GTCCAGCTCGCTCTACAACA-3′; *Slc2a4*, forward 5′-ATCATCCGGAACCTGGAGG-3′, reverse 5′-CGGTCAGGCGCTTTAGACTC-3′; *Pfkm*, forward 5′-GGGACACCATCAGCCTTTGA-3′, reverse 5′-TCCCCTCCAAAAGTGCCATC-3′; *Gys1*, forward 5′-CGAATCCCTTTTAGTGGGGAG-3′, reverse 5′-TGAGGAGTCGTCCAGCATGT-3′; *Rn18s*, forward 5′-GCAATTATTCCCCATGAACG-3′, reverse 5′-GGCCTCACTAAACCATCCAA-3′.

### 2-deoxy-D-[1,2-^3^H]-glucose uptake

Cells were seeded in 12-well dishes and synchronised for 48 h in 1% FBS. Cells were then given low 5.5 mM or 25 mM glucose DMEM 10% FBS supplemented with 0.2 µCi/ml and 0.9 µCi/ml 2-deoxy-D-[1,2-^3^H]-glucose, respectively (PerkinElmer). At the indicated time points cell culture supernatants were collected, and cell layers were washed once with PBS and lysed with 0.1 N NaOH. Radioactivity was measured using a Beckmann LS 6500 scintillation counter, quench corrected and normalised to protein content, as determined by Bradford assay.

### Real-time bioenergetics

For real-time determination of the oxygen consumption rate (OCR) and extracellular acidification rate (ECAR), a Seahorse XFe96 Analyzer was used (Agilent Technologies). 24 h in advance, cells were seeded in 5.5 mM glucose DMEM with 10% FBS at 20,000 cells per well in a XF96 Seahorse plate. 1 h before measurements, cells were given XF base media supplemented with 2 mM glutamine, 1 mM pyruvate, 10% FBS and either 5.5 mM glucose or 25 mM glucose. OCR and ECAR measurements were collected for 90 min to achieve a baseline, after which insulin was injected to a final concentration of 100 nM and measurements were taken for another 90 min. For mitochondrial substrate dependency, a Seahorse XF Mito Fuel Flex Test Kit was used to manufacturer’s specifications. Experiments were conducted using media without serum, which was shown to interfere with inhibitor efficacy in a pilot run. Pathway dependency was calculated with Wave Software (Agilent Technologies), where Dependency % = ((baseline OCR − target inhibitor OCR)/(baseline OCR − all inhibitors OCR)) × 100.

### Statistical analysis

Statistical analyses were performed using the GraphPad Prism Software Version 7.0 (GraphPad Software). All data are presented as a mean ± standard error of the mean (SEM), and outliers identified by Grubb’s test (α = 0.05) were excluded. For cardiac fibroblast cultures and animals used for experimental obesity and insulin resistance, each “n” represents a distinct biological replicate. For KYSE cell culture, each “n” represents a distinct passage. Unpaired t-test was used to compare groups of two; otherwise, ANOVA with Sidak’s multiple-comparison correction was used. *P* values of < 0.05 were considered statistically significant. *P ≤ 0.05, **P ≤ 0.005, ***P ≤ 0.0005, ****P ≤ 0.0001.

## Electronic supplementary material

Supplemental information

## Data Availability

The datasets generated in the current study are available from the corresponding author upon reasonable request.

## References

[1] Cavender MA (2015). Impact of Diabetes Mellitus on Hospitalization for Heart Failure, Cardiovascular Events, and Death: Outcomes at 4 Years From the Reduction of Atherothrombosis for Continued Health (REACH) Registry. Circulation.

[2] MacDonald MR (2008). Impact of diabetes on outcomes in patients with low and preserved ejection fraction heart failure: an analysis of the Candesartan in Heart failure: Assessment of Reduction in Mortality and morbidity (CHARM) programme. Eur Heart J.

[3] van Heerebeek L (2008). Diastolic stiffness of the failing diabetic heart: importance of fibrosis, advanced glycation end products, and myocyte resting tension. Circulation.

[4] Ivey MJ, Tallquist MD (2016). Defining the Cardiac Fibroblast. Circulation journal: official journal of the Japanese Circulation Society.

[5] Sandanger O (2013). The NLRP3 inflammasome is up-regulated in cardiac fibroblasts and mediates myocardial ischaemia-reperfusion injury. Cardiovasc Res.

[6] Woodall MC, Woodall BP, Gao E, Yuan A, Koch WJ (2016). Cardiac Fibroblast GRK2 Deletion Enhances Contractility and Remodeling Following Ischemia/Reperfusion Injury. Circ Res.

[7] Fiaschi T (2014). Hyperglycemia and angiotensin II cooperate to enhance collagen I deposition by cardiac fibroblasts through a ROS-STAT3-dependent mechanism. Biochim Biophys Acta.

[8] Tang M (2007). High glucose promotes the production of collagen types I and III by cardiac fibroblasts through a pathway dependent on extracellular-signal-regulated kinase 1/2. Mol Cell Biochem.

[9] Zhou H (2011). Fasudil hydrochloride hydrate, a Rho-kinase inhibitor, suppresses high glucose-induced proliferation and collagen synthesis in rat cardiac fibroblasts. Clin Exp Pharmacol Physiol.

[10] Singh VP, Baker KM, Kumar R (2008). Activation of the intracellular renin-angiotensin system in cardiac fibroblasts by high glucose: role in extracellular matrix production. Am J Physiol Heart Circ Physiol.

[11] Kleeschulte S, Jerrentrup J, Gorski D, Schmitt J, Fender AC (2018). Evidence for functional PAR-4 thrombin receptor expression in cardiac fibroblasts and its regulation by high glucose: PAR-4 in cardiac fibroblasts. Int J Cardiol.

[12] Zhang X (2018). H3 relaxin inhibits the collagen synthesis via ROS- and P2X7R-mediated NLRP3 inflammasome activation in cardiac fibroblasts under high glucose. J Cell Mol Med.

[13] Muller J (2014). Interleukin-6-dependent phenotypic modulation of cardiac fibroblasts after acute myocardial infarction. Basic Res Cardiol.

[14] Midgley AC (2013). Transforming growth factor-beta1 (TGF-beta1)-stimulated fibroblast to myofibroblast differentiation is mediated by hyaluronan (HA)-facilitated epidermal growth factor receptor (EGFR) and CD44 co-localization in lipid rafts. J Biol Chem.

[15] Li Y (2011). Severe lung fibrosis requires an invasive fibroblast phenotype regulated by hyaluronan and CD44. The Journal of experimental medicine.

[16] Meran S (2011). Hyaluronan facilitates transforming growth factor-beta1-dependent proliferation via CD44 and epidermal growth factor receptor interaction. J Biol Chem.

[17] Heickendorff L, Ledet T, Rasmussen LM (1994). Glycosaminoglycans in the human aorta in diabetes mellitus: a study of tunica media from areas with and without atherosclerotic plaque. Diabetologia.

[18] Bogdani M (2014). Hyaluronan and hyaluronan-binding proteins accumulate in both human type 1 diabetic islets and lymphoid tissues and associate with inflammatory cells in insulitis. Diabetes.

[19] Papanastasopoulou, C. *et al*. Cardiovascular Risk and Serum Hyaluronic Acid: A Preliminary Study in a Healthy Population of Low/Intermediate Risk. *Journal of clinical laboratory analysis* **31**, 10.1002/jcla.22010 (2017). 10.1002/jcla.22010 PMC6816791 27306798

[20] Ren J, Hascall VC, Wang A (2009). Cyclin D3 mediates synthesis of a hyaluronan matrix that is adhesive for monocytes in mesangial cells stimulated to divide in hyperglycemic medium. J Biol Chem.

[21] Kang L (2013). Hyaluronan accumulates with high-fat feeding and contributes to insulin resistance. Diabetes.

[22] Nagy N (2015). Inhibition of hyaluronan synthesis restores immune tolerance during autoimmune insulitis. The Journal of clinical investigation.

[23] Jones S, Jones S, Phillips AO (2001). Regulation of renal proximal tubular epithelial cell hyaluronan generation: implications for diabetic nephropathy. Kidney international.

[24] Takeda M, Babazono T, Nitta K, Iwamoto Y (2001). High glucose stimulates hyaluronan production by renal interstitial fibroblasts through the protein kinase C and transforming growth factor-beta cascade. Metabolism: clinical and experimental.

[25] Yevdokimova NY (2003). High glucose-induced alterations of extracellular matrix of human skin fibroblasts are not dependent on TSP-1-TGFbeta1 pathway. Journal of diabetes and its complications.

[26] Sainio A, Jokela T, Tammi MI, Jarvelainen H (2010). Hyperglycemic conditions modulate connective tissue reorganization by human vascular smooth muscle cells through stimulation of hyaluronan synthesis. Glycobiology.

[27] Yevdokimova NY, Komisarenko SV (2003). The influence of high ambient glucose level on the production of pericellular glycosaminoglycans by cultured endothelial cells. Ukrains’kyi biokhimichnyi zhurnal (1999).

[28] Wang A, Hascall VC (2004). Hyaluronan structures synthesized by rat mesangial cells in response to hyperglycemia induce monocyte adhesion. J Biol Chem.

[29] Twarock S (2017). Hyperglycaemia and aberrated insulin signalling stimulate tumour progression via induction of the extracellular matrix component hyaluronan. International journal of cancer.

[30] Hascall VC (2014). The dynamic metabolism of hyaluronan regulates the cytosolic concentration of UDP-GlcNAc. Matrix Biol.

[31] Vigetti D (2014). Natural antisense transcript for hyaluronan synthase 2 (HAS2-AS1) induces transcription of HAS2 via protein O-GlcNAcylation. J Biol Chem.

[32] Becker C (2001). The endosomal compartment is an insulin-sensitive recruitment site for GLUT4 and GLUT1 glucose transporters in cardiac myocytes. Endocrinology.

[33] Kolwicz, S. C., Purohit, S. & Tian, R. Cardiac Metabolism and Its Interactions with Contraction, Growth, and Survival of the Cardiomyocte. *Circulation research* **113**, 10.1161/CIRCRESAHA.113.302095 (2013). 10.1161/CIRCRESAHA.113.302095 PMC3845521 23948585

[34] Ale-Agha N (2018). CDKN1B/p27 is localized in mitochondria and improves respiration-dependent processes in the cardiovascular system-New mode of action for caffeine. PLoS biology.

[35] Alex, L., Russo, I., Holoborodko, V. & Frangogiannis, N. G. Characterization of a mouse model of obesity-related fibrotic cardiomyopathy that recapitulates features of human Heart Failure with Preserved Ejection Fraction. *Am J Physiol Heart Circ Physiol*, 10.1152/ajpheart.00238.2018 (2018). 10.1152/ajpheart.00238.2018 PMC6230908 30004258

[36] Hutchinson KR, Lord CK, West TA, Stewart JA (2013). Cardiac fibroblast-dependent extracellular matrix accumulation is associated with diastolic stiffness in type 2 diabetes. PLoS One.

[37] Russo I, Frangogiannis NG (2016). Diabetes-associated cardiac fibrosis: Cellular effectors, molecular mechanisms and therapeutic opportunities. J Mol Cell Cardiol.

[38] Urbina P, Singla DK (2014). BMP-7 attenuates adverse cardiac remodeling mediated through M2 macrophages in prediabetic cardiomyopathy. Am J Physiol Heart Circ Physiol.

[39] Fukuda M (2010). Potentiation by candesartan of protective effects of pioglitazone against type 2 diabetic cardiovascular and renal complications in obese mice. Journal of hypertension.

[40] Tokudome T (2004). Direct effects of high glucose and insulin on protein synthesis in cultured cardiac myocytes and DNA and collagen synthesis in cardiac fibroblasts. Metabolism: clinical and experimental.

[41] Zhang Y (2016). Deletion of interleukin-6 alleviated interstitial fibrosis in streptozotocin-induced diabetic cardiomyopathy of mice through affecting TGFbeta1 and miR-29pathways. Scientific reports.

[42] Prata C (2017). Glycosides from Stevia rebaudiana Bertoni Possess Insulin-Mimetic and Antioxidant Activities in Rat Cardiac Fibroblasts. Oxidative medicine and cellular longevity.

[43] Ritterhoff J, Tian R (2017). Metabolism in cardiomyopathy: every substrate matters. Cardiovasc Res.

[44] Lopaschuk GD, Spafford MA (1990). Energy substrate utilization by isolated working hearts from newborn rabbits. The American journal of physiology.

[45] Lopaschuk GD, Spafford MA, Marsh DR (1991). Glycolysis is predominant source of myocardial ATP production immediately after birth. The American journal of physiology.

[46] Wang C, Hu SM (1991). Developmental regulation in the expression of rat heart glucose transporters. Biochemical and biophysical research communications.

[47] Jokela TA (2008). Induction of hyaluronan cables and monocyte adherence in epidermal keratinocytes. Connective tissue research.

[48] Zhuang Y, Yin Q (2013). Peroxisome proliferator-activated receptor gamma agonists attenuate hyperglycaemia-induced hyaluronan secretion in vascular smooth muscle cells by inhibiting PKCbeta2. Cell biochemistry and biophysics.

[49] Siiskonen H (2014). Hyaluronan synthase 1 (HAS1) produces a cytokine-and glucose-inducible, CD44-dependent cell surface coat. Exp Cell Res.

[50] Pahwa R, Nallasamy P, Jialal I (2016). Toll-like receptors 2 and 4 mediate hyperglycemia induced macrovascular aortic endothelial cell inflammation and perturbation of the endothelial glycocalyx. Journal of diabetes and its complications.

[51] Parker GJ, Lund KC, Taylor RP, McClain DA (2003). Insulin resistance of glycogen synthase mediated by o-linked N-acetylglucosamine. J Biol Chem.

[52] Marshall S, Bacote V, Traxinger RR (1991). Discovery of a metabolic pathway mediating glucose-induced desensitization of the glucose transport system. Role of hexosamine biosynthesis in the induction of insulin resistance. J Biol Chem.

[53] Zachara NE, Hart GW (2004). O-GlcNAc a sensor of cellular state: the role of nucleocytoplasmic glycosylation in modulating cellular function in response to nutrition and stress. Biochim Biophys Acta.

[54] Wang A, Ren J, Wang CP, Hascall VC (2014). Heparin prevents intracellular hyaluronan synthesis and autophagy responses in hyperglycemic dividing mesangial cells and activates synthesis of an extensive extracellular monocyte-adhesive hyaluronan matrix after completing cell division. J Biol Chem.

[55] Neumann S (2002). Aldosterone and D-glucose stimulate the proliferation of human cardiac myofibroblasts *in vitro*. Hypertension.

[56] Hohl M (2013). HDAC4 controls histone methylation in response to elevated cardiac load. The Journal of clinical investigation.

[57] Dirkx E, da Costa Martins PA, De Windt LJ (2013). Regulation of fetal gene expression in heart failure. Biochim Biophys Acta.

[58] Bertero, E. & Maack, C. Metabolic remodelling in heart failure. *Nature reviews. Cardiology*, 10.1038/s41569-018-0044-6 (2018). 10.1038/s41569-018-0044-6 29915254

[59] Young LH (1997). Low-flow ischemia leads to translocation of canine heart GLUT-4 and GLUT-1 glucose transporters to the sarcolemma *in vivo*. Circulation.

[60] Midura RJ, Cali V, Lauer ME, Calabro A, Hascall VC (2018). Quantification of hyaluronan (HA) using a simplified fluorophore-assisted carbohydrate electrophoresis (FACE) procedure. Methods Cell Biol.

[61] Bookout, A. L., Cummins, C. L., Mangelsdorf, D. J., Pesola, J. M. & Kramer, M. F. In *Current Protocols in Molecular Biology* (John Wiley & Sons, Inc., 2001).

