# Supplementary material for: Cardiac fibroblast activation and hyaluronan synthesis in response to hyperglycemia and diet-induced insulin resistance
Source: Sci Rep. 2019 Feb 12;9:1827. doi: 10.1038/s41598-018-36140-6 (PMC6372628; doi:10.1038/s41598-018-36140-6)
Supplement: Supplementary file 1 — Supplemental information [file 41598_2018_36140_MOESM1_ESM.pdf]

SUPPLEMENTAL INFORMATION

Cardiac fibroblast activation and hyaluronan synthesis in response to hyperglycemia and diet-induced insulin resistance

Daniel J Gorski [1], Anne Petz [1], Christina Reichert [1], Sören Twarock [1], Maria Grandoch [1], Jens W. Fischer [1,*]

1-Institute of Pharmacology and Clinical Pharmacology, University Clinic, Heinrich-Heine-University Duesseldorf, Duesseldorf, Germany

*Correspondence to Jens W. Fischer:
Institut für Pharmakologie und Klinische Pharmakologie
Universitätsklinikum der Heinrich-Heine-Universität Düsseldorf
Moorenstr. 5
40225 Düsseldorf
Germany
Phone: +49 211 81 12500
Fax: +49 211 81 14781
E-Mail: jens.fischer@uni-duesseldorf.de

SUPPLEMENTAL FIGURES

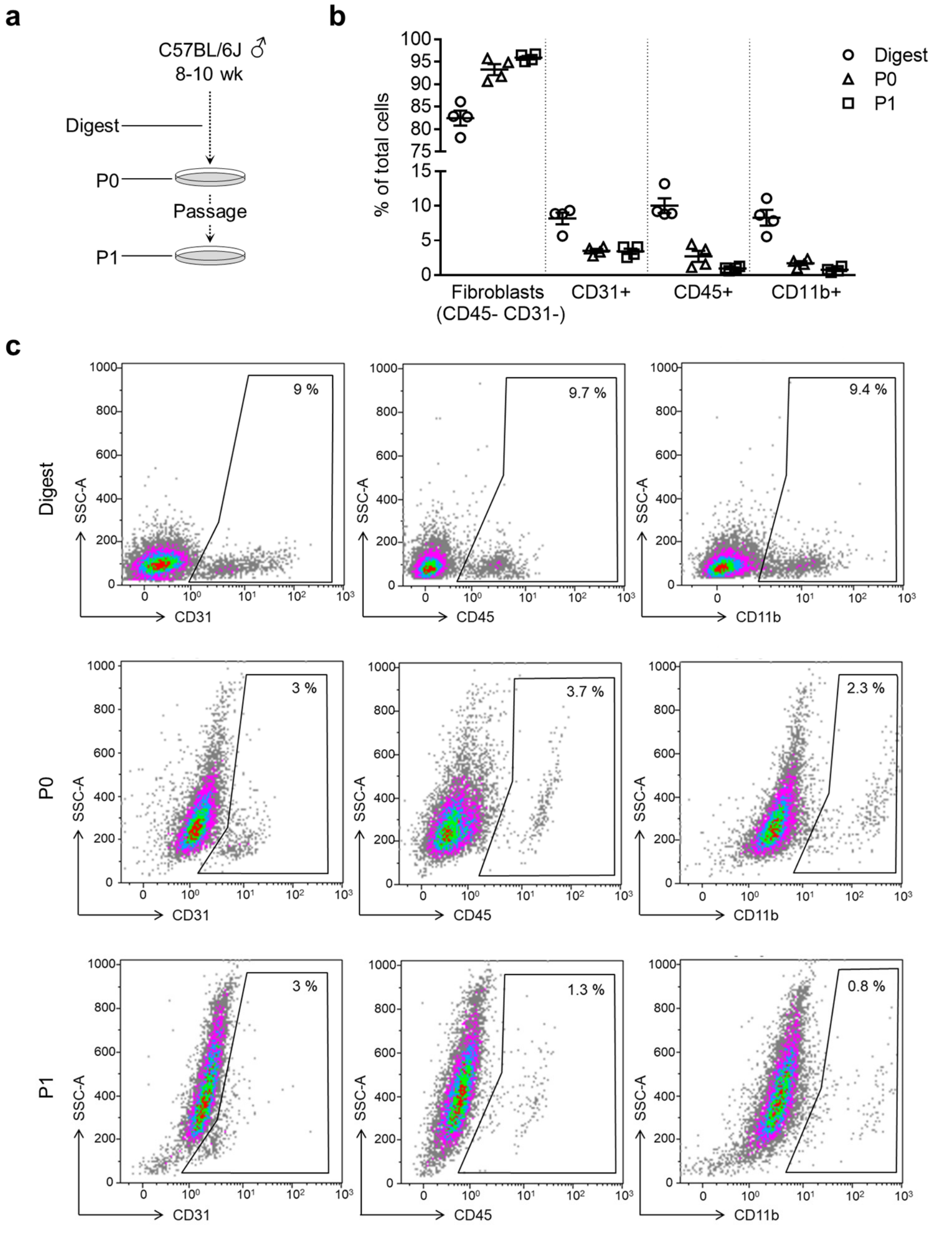

Supplemental Fig. 1. *Cardiac fibroblast culture purity assessed by flow cytometry.* To determine cellular heterogeneity in cardiac fibroblast cell cultures, contaminating endothelial cells (CD31+), leucocytes (CD45+) and myeloid cells (CD11b+) were quantified at each step of cell culture. This included the total cardiac digest (Digest), adherent cells 96 h after initial plating (P0) and cells after one trypsin passage (P1). Experiments were conducted in P1, in which cardiac fibroblasts were highly enriched to 96%. **a**, Experimental schematic. **b**, Quantification of cell types represented as the % of total cells at each cell culture step (n=4). **c**, Representative flow cytometric plots (%) indicates % of total cells. Data represent mean ± SEM.

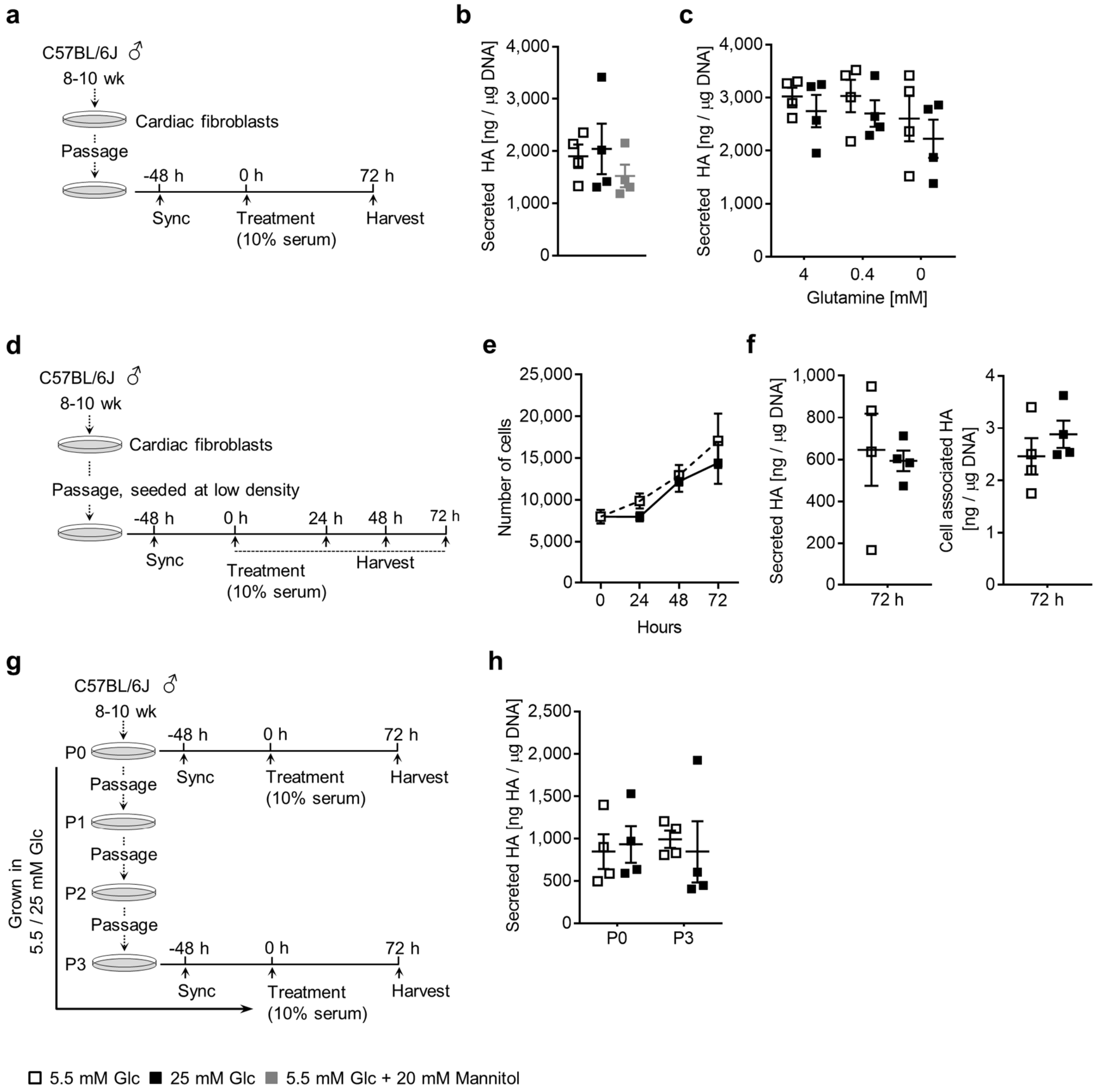

Supplemental Fig. 2. *Various cell culture methods do not alter HA matrix formation in response to hyperglycemia.* Primary cardiac fibroblast cultures were treated with media containing 5.5 or 25 mM glucose in 10% FBS for up to 72 hours, using modified cell culture methods to determine the contributions of osmotic pressure, glutamine abundance, active proliferation and chronic exposure to hyperglycemia during passage to HA matrix formation. **a***,* Schematic of experimental design to test osmotic pressure and glutamine concentration (b, c). **b**, **c**, Quantification of HA secretion into the media (n=4). **d***,* Schematic of experimental design to test actively proliferating cells (e, f). **e**, Proliferation (n=7). **f**, Quantification of HA secretion into the media and cell layer (n=4). **g***, S*chematic of experimental design to test for early and chronic exposure to hyperglycemia (h). **h**, Quantification of HA secretion into the media and cell layer (n=4). Data represent mean ± SEM. One-way ANOVA with Sidak's multiple-comparison correction (b, c, h), two-way ANOVA with Sidak's multiple-comparison correction (e) and unpaired t-test (f)**.**

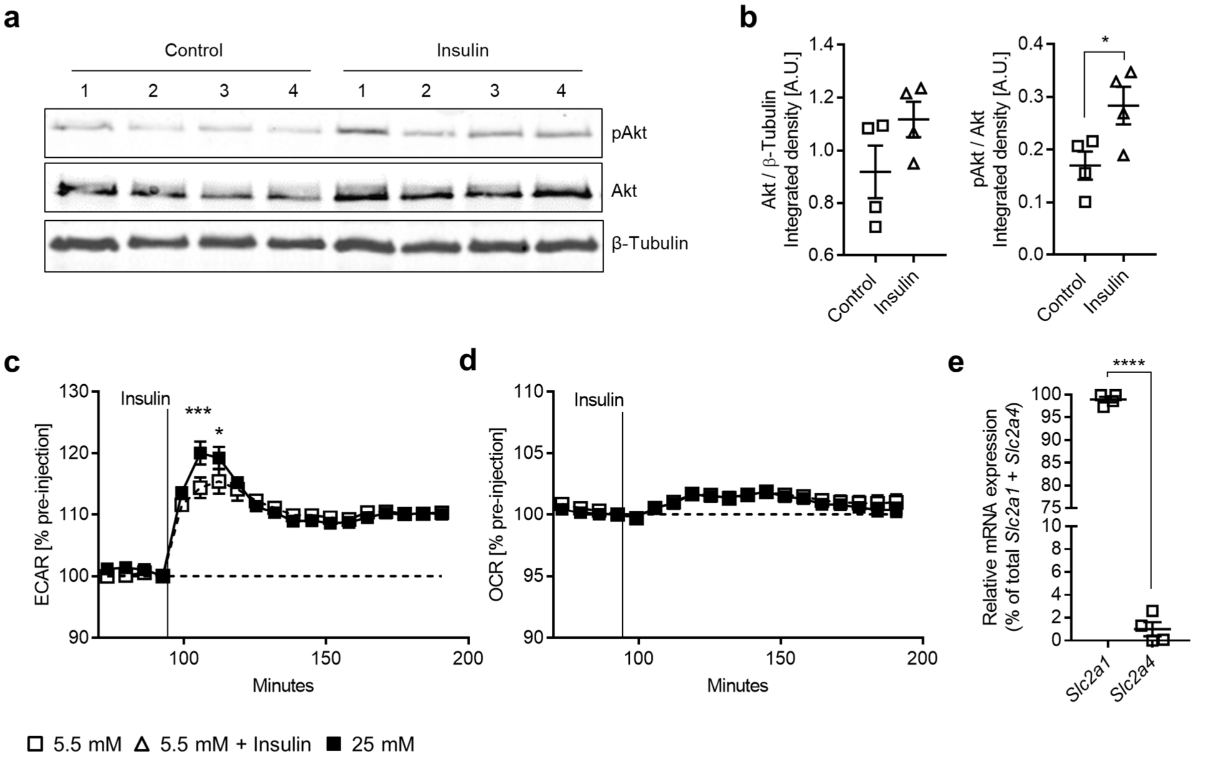

Supplemental Fig. 3. *Acute response to insulin stimulation.* **a,** Immunoblot of pAkt, Akt and β-tubulin (re-probe of pAkt) after 5 min incubation with 100 nM insulin from 4 distinct cultures (1-4). Image grouping taken from different parts of the same blot for pAkt and β-tubulin and a different blot for Akt. Images were cropped to display the area of interest (n=4). **b**, Quantification of bands in (a). **c**, ECAR time course of cardiac fibroblasts given 100 nM insulin, relative to pre-injection ECAR baseline (n=4). **d**, OCR time course of cardiac fibroblasts given 100 nM insulin, relative to pre-injection OCR baseline (n=4). **e,** Quantification of *Slc2a1* (GLUT1) and *Slc2a4* (GLUT4) mRNA expression after 72 h. Primer efficiency corrected and displayed as the relative expression of the total of *Slc2a1* + *Slc2a4* (n=4). For analysis of mRNA expression *Rn18S* was used as an internal control. Data represent mean ± SEM; unpaired t-test (b, e)**,** two-way ANOVA with Sidak's multiple-comparison correction (c, d). *$P \leq 0.05$, ***$P \leq 0.0005$, ****$P < 0.0001$.

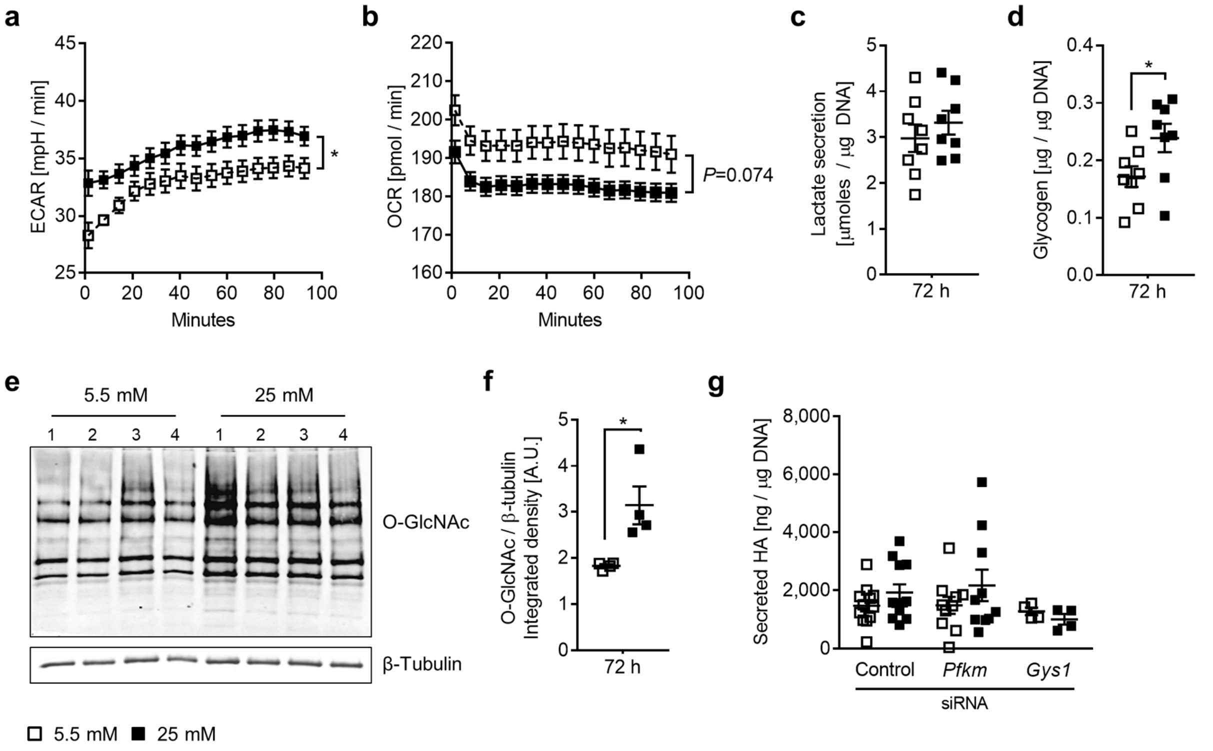

Supplemental Fig. 4. *Acute hyperglycemia alters glucose utilisation in cardiac fibroblasts.* Primary cardiac fibroblast cultures were treated with media containing 5.5 or 25 mM glucose for up to 72 hours. **a**, ECAR time course and **b**, OCR time course determined after acute exposure (1 h) to hyperglycemia (n=4). **c**, Quantification of lactate in the media after 72 h (n=8). **d**, Quantification of glycogen content after 72 h (n=8). **e**, Immunoblot of O-GlcNAcylated proteins in cell extracts isolated after 72 h (anti-O-linked N-acetylglucosamine, RL2), re-probed with anti-β-tubulin for loading control, four separate cultures (1-4). Image grouping was cropped from different parts of the same blot to display areas of interest (n=4). **f**, Quantification of (e). **g**, Quantification of secreted HA (72 h) after siRNA knockdown of *Pfkm* (n=10), *Gys1* (n=4) or scramble control (n=12). Data represent mean ± SEM; two-way ANOVA with Sidak's multiple-comparison correction (a, b), unpaired t-test (c, d, f) and one-way ANOVA with Sidak’s multiple-comparison correction (g). *$P \leq 0.05$.

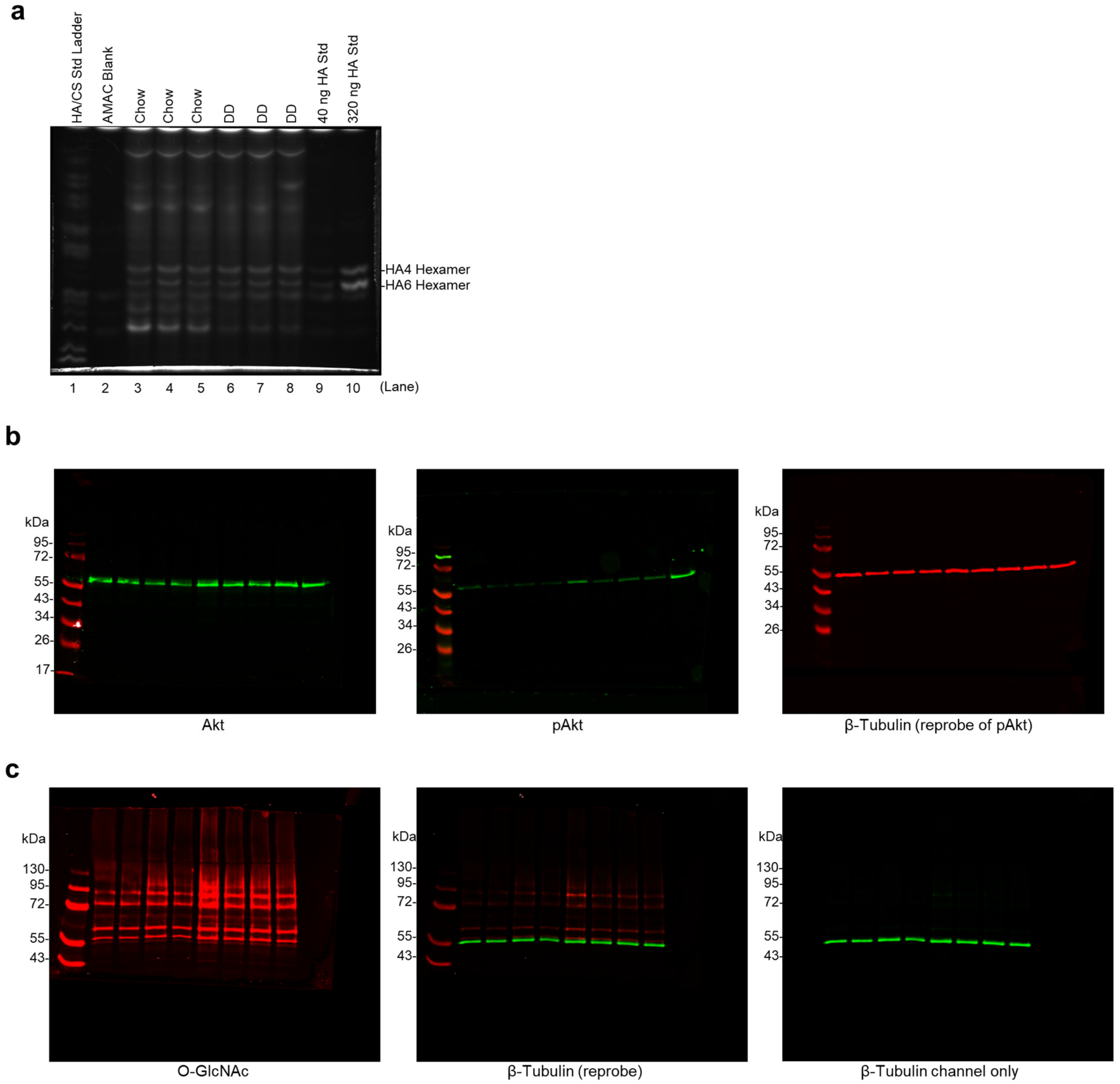

Supplemental Fig. 5. *Full-length gels*. **a**, Full-length gel of cardiac HA analysis by FACE. Lanes 5 and 6 were cropped; color was inverted and displayed as figure 6b. **b**, Full-length immunoblots displayed as cropped images in supplemental figure 3a. Lane loading from left to right: MW marker, control-1, control-2, control-3, control-4, insulin-1, insulin-2, insulin-3, insulin-4 and 10% FBS positive control. **c**, Full-length immunoblots displayed as cropped images in supplemental figure 4e. Lane loading from left to right: MW marker, 5.5 mM-1, 5.5 mM-2, 5.5 mM-3, 5.5 mM-4, 25 mM-1, 25 mM-2, 25 mM-3 and 25 mM-4.

SUPPLEMENTAL METHODS

Flow Cytometry

Heart digests were filtered and centrifuged at 300 x *g* for 10 min, and the pellet was resuspended and incubated for 30 min at 37° C in RPMI medium (Gibco Life Technologies). In the case of adherent cells (P0, P1), cells were harvested with Trypsin-EDTA, pelleted and incubated for 30 min at 37° C in RPMI medium. The single cell suspensions were incubated with purified anti-mouse CD16/32 antibody (Biolegend); LIVE/DEAD™ Fixable Aqua Dead Cell Stain Kit (Life Technologies) and a combination of antibodies to identify leucocytes: CD45 – AF700, clone30-F11 (BioLegend), myeloid cells, CD11b – PE, clone M1/70 (BD Bioscience) and endothelial cells, CD31 – APC, clone 390, (BioLegend). Measurements were performed with a LSRII flow cytometer (BD Bioscience). Kaluza Flow Analysis Software (Beckman Coulter) was used for subsequent data analysis.

Lactate and glycogen quantification

Measurement of lactate in cell culture supernatants and intracellular glycogen was performed with fluorometric-based assay kits (Lactate Assay Kit, Sigma-Aldrich, MAK064 and Glycogen Assay Kit, Abcam, ab65620) according to the manufacturers' instructions. Values were normalised to DNA content, determined by the Quant-iT PicoGreen dsDNA Assay Kit (Molecular Probes).

Immunoblotting

Cells were lysed in laemmli sample (O-GlcNAc blots) or RIPA (Akt blots) buffer and separated by 8% Tris-Glycine SDS-PAGE. Gels were blotted onto nitrocellulose membranes and probed with anti-O-linked N-acetylglucosamine (O-GlcNAc) antibody [RL2] (Abcam, ab2739), anti-pAkt (Cell Signalling Phospho-Akt (Ser473) (D9E) XP Rabbit mAb #4060) or anti-Akt (Akt (pan) (C67E7) Rabbit mAb #4691), then re-probed with anti-β-tubulin I antibody (Sigma-Aldrich, T7816). Goat anti-mouse IRDye800CW, goat anti-rabbit IRDye800CW and goat anti-mouse IRDye680RD (LI-COR Biosciences) were used as secondary antibodies. Signals were detected with the Odyssey infrared imaging system (LI-COR Biosciences) and quantified using VisionWorks software (Analytik Jena AG).

RNA interference

Cells were transfected using Lipofectamine RNAiMAX (Life Technologies) according to the manufacturer's instructions. Cultures were incubated with siRNAs (Qiagen) for 24 h, then synchronised for 48 h in 1% FBS, followed by treatment. The following siRNA catalogue numbers were used; *Pfkm* (SI01375808), *Gys1* (SI04417609) and AllStars Negative Control (SI03650318). Quantitative real-time RT-PCR was performed to confirm knockdown efficiency; only samples with > 50% knockdown were used.
